# Muscle Strength and Hamstrings to Quadriceps Ratio in Young Soccer Players: A Cross-Sectional Study

**DOI:** 10.3390/jfmk8020070

**Published:** 2023-05-23

**Authors:** Athanasios Mandroukas, Yiannis Michailidis, Thomas Metaxas

**Affiliations:** Laboratory of Evaluation of Human Biological Performance, Department of Physical Education and Sport Sciences, Aristotle University of Thessaloniki, 57001 Thessaloniki, Greece; ioannimd@phed.auth.gr

**Keywords:** isokinetic muscle strength, soccer, H:Q ratio, developmental ages

## Abstract

The aim of the present study was to examine and compare the concentric isokinetic peak torque of the knee flexors and extensors muscles, as well as their ratio, in young soccer players. Two hundred and sixty-five (n = 265) young soccer players were divided into five groups: U-12 (n = 43, mean age 11.5 ± 0.4 yrs), U-14 (n = 63, mean age 13.6 ± 0.3 yrs), U-16 (n = 64, mean age 15.4 ± 0.5 yrs), U-18 (n = 53, mean age 17.5 ± 0.4 yrs) and U-20 (n = 42, mean age 19.3 ± 0.6 yrs). Three maximal voluntary isokinetic leg extensions and flexions at angular velocities of 60, 180, and 300°·s^−1^, and H:Q strength ratio was determined. The largest H:Q strength ratio for all ages, with the exception of age group U-12, appears at a slow angular velocity of 60°·s^−1^, and the smallest H:Q ratio at a fast angular velocity of 300°·s^−1^. In age group U-12, at an angular velocity of 60°·s^−1^, the strength of the quadriceps muscle was almost twice the strength of the hamstrings. The H:Q strength ratio was smaller in age group U-12 and greater in group U-20. In age group U-12, the greatest H:Q strength ratio appeared at an angular velocity of 180°·s^−1^, while in the other age groups, it appeared at 60°·s^−1^. Strength training of hamstring muscles remains inadequate across ages. The small H:Q strength ratio in younger ages and the large H:Q ratio in older ages suggest that high-intensity training may increase the H:Q strength ratio, which, in turn, may protect the knee joint from excessive and burdensome loads.

## 1. Introduction

Soccer has become very popular over the last two decades. It is a complex sport that involves many activities, such as tackles, jumps, directional and speed changes, feints, and kicking [1]. These activities place great stress on the muscles and joints of the lower limbs; therefore, the development of strength in soccer players is very important [2].

The knee, the largest and perhaps most complex joint in the body, is a two-joint structure composed of the tibiofemoral joint and the patellofemoral joint. The hamstring and quadriceps are two postural muscles with antagonistic actions. The hamstring muscle comprises spindle muscles that have a complex anatomy. What is more, the function of the four individual muscle portions, three of which span both the knee and hip joint, is not fully understood [3]. The hamstrings act, in particular, to extend and flex the hip joint. They participate in many types of locomotion, including decelerating activities involving eccentric contractions [4]. Accordingly, the quadriceps muscle is formed by four muscles that extend the knee and, accessorily, the thigh. This last movement is carried out by the bi-articular rectus femoris. The quadriceps plays an important role in jumping and ball kicking [5].

The quadriceps and hamstring muscles have different structural, functional, and metabolic characteristics. Compared to the quadriceps, the hamstring muscle has a greater proportion of fast-twitch (type II) muscle fibers [6], a smaller total cross-sectional area [7], and a smaller muscle mass [8]. A main characteristic of the fast-twitch fibers is that they have a greater capacity to generate power per unit of cross-sectional area [9] compared to the slow-twitch (type I) muscle fibers. Based on the existing information about adult soccer players [1] as well as on data acquired from sixteen-year-old athletes of various sports [10], the vastus lateralis and the quadriceps muscles are characterized as mixed-fiber type muscles [11]. Imbalance between the knee flexors and extensors has been traditionally assessed by the conventional concentric hamstrings:quadriceps ratio (Hcon:Qcon), calculated as the maximal concentric knee flexion strength divided by the maximal concentric knee extension strength at the same angular velocity [12,13,14,15,16]. Former studies conducted on adults estimated that the minimum ratio of conventional Hcon:Qcon should be 0.6, namely, the hamstring should be 60% as strong as the quadriceps [13,17], for preventing hamstring and/or knee-related injuries [18,19]. Accordingly, it has been supported that the functional or mixed ratio of maximal eccentric knee flexion to maximal concentric knee extension, hamstring eccentric:quadriceps concentric (Hecc:Qcon) should be close to or above 1.0 [17,20] for preventing anterior cruciate ligament (ACL) and hamstring injuries because forces produced during eccentric muscle actions are substantially greater than those produced during concentric muscle actions [21,22], particularly at higher velocities [23].

The H:Q conventional ratio is the oldest and most examined parameter for the strength relationship of the hamstrings and quadriceps muscles. Previous studies have reported results ranging from 50 to 83% [20,24]. Many studies have examined isokinetic muscle strength in different angular velocities, sports, and types of training [25,26,27]; however, the number of studies with a large sample size in the developmental ages is very limited.

In adult male soccer players, isokinetic strength has been studied extensively [28,29,30]; however, less information is available about the development of muscle strength and H:Q ratio in children. Therefore, the purpose of the present study was to examine and compare the concentric isokinetic absolute and relative peak torque of the knee flexor and knee extensor muscles at a variety of angular velocities, as well as their ratio, in a large number of young soccer players throughout the developmental years, namely from age 12 to age 20. We hypothesized that a significant increase in hamstring and quadriceps strength occurs as an effect of chronological age, whereas the H:Q strength ratio remains unaltered or changes with increased angular velocity.

## 2. Materials and Methods

### 2.1. Subjects

The power analysis was conducted prior to the study being performed based on previous studies of similar research design. An effect size of >0.3, a probability error of 0.05, and a power of 0.95 were used for the five groups. Those indicated that 215 subjects was the smallest acceptable number of participants to analyze the interaction. The calculations for effect size (ES) and statistical power were performed with G*Power software: Statistical Power Analyzes for Windows, Version 3.1.9.7, according to Cohen’s f criteria [31,32].

A total of 265 (n = 265) young soccer players throughout the developmental years of 12–20 participated in this study. The participants were divided into five groups. The physical characteristics of the subjects are shown in Table 1. The leg used most frequently for kicking the ball was identified as the dominant leg. All participants were asked to visit the laboratory several times prior to the beginning of the experiment in order to become familiar with the surroundings as well as the procedures to be followed. To minimize the occurrence of systematic measurement errors, the participants were verbally instructed with language adapted to their age. Tanner’s stages were not examined in these participants.

Before the start of the study, written consent from the players and their parents was obtained following a full explanation of the procedures involved. The subjects were familiarized by performing several submaximal contractions with the testing procedures as well as with the investigators before the measurements. None of the participants reported musculoskeletal injuries of the lower limbs that would prevent them from performing maximal exercise. The exclusion criteria were: recent history of muscle injury of a lower limb, present complaint of thigh and leg pain, or any other medical problems contraindicated to experimental testing. None of the subjects had been doing progressive resistive exercise the day before testing, and their sleep pattern was sufficient (6–8 h) in order to arrive to the laboratory in a rested condition. The participants were a highly selective group with regard to skills, performance, size, anthropometric characteristics, and physical condition. This study has been approved by the Institutional Review Board of the Exercise Physiology and Sport Rehabilitation Laboratory, Thessaloniki, Greece (No. 03/2020), and was in accordance with the Declaration of Helsinki.

All participants in the study came from the same football academy, which followed a consistent training program across the age groups. The duration of the training season for the 12- and 14-year-old athletes was 44 weeks, while for the 16–20-year-olds, it was 46 weeks per year. In particular, the 12-year-old subjects systematically performed three training sessions per week of 75 min each, the 14-year-olds performed four training sessions per week of 90 min each, and the 16–20-year-old athletes participated in five training sessions of 90 min each per week. Furthermore, all players had one additional specific personal training session every 15 days for individual improvement in certain skills (technical elements and conditioning). In total, the annual number of training sessions for the 12- and 14-year-olds was 130 and 170, respectively, while for the 16–20-year-old age groups it was 220. Moreover, all players competed in one game per week throughout the season. Training in prepubertal and pubertal age (12–16 years old), in general, aims at creating versatile players by developing physical performance through specific exercises tailored to the sport of soccer and the age level. The training protocol was based on the normal technical–tactical and physiological elements of soccer indicated for improvement, according to developmental age. In the 18- and 20-year-old age groups, where body weight and height have stabilized, the workout is adapted to the training regimen of first competitive category players. Training at these ages is taking place on a daily basis and is more intensive and more systematic, and weight training is a prerequisite for increasing muscle strength. No athlete followed a personalized weight training routine. All players followed the soccer training program designed by their academy.

### 2.2. Isokinetic Strength Testing

The strength of knee flexors and extensors in the dominant leg was measured using a Cybex II isokinetic dynamometer (Lumex Inc., Ronkonkoma, New York, NY, USA). Prior to each testing session, participants performed a 10 min warm-up on a cycle ergometer (Monark 839, Varberg, Sweden) followed by a 5 min partial passive stretch of the knee flexors and extensors [33], and the unilateral concentric muscle strength of the dominant leg was measured on the isokinetic dynamometer. For each angular velocity, peak isokinetic torque was recorded simultaneously, and the torque generated by the limb weight and the dynamometer arm was extracted from the obtained data. Afterwards, each subject was asked to sit on the dynamometer in an adjustable chair; the upper body was stabilized with straps secured diagonally across the chest and around the hips and thighs to prevent any extraneous joint movement. The position chosen for the knee to be examined was at 90° of flexion (0° corresponding to a fully extended knee) so as to be in alignment with the axis of the dynamometer lever arm with the distal point of the lateral femoral condyle. The length of the level arm was regulated separately for each participant, and the resistance pad was set proximal to the medial malleolus. The other leg was hanging freely, without any forces exerted on it. The knee movements were consecutive: extension was performed when the knee was at a 90° flexed position, and flexion was performed when the knee was fully extended at 0°. Participants were asked to kick their tested leg with as much strength and speed as they could through a complete range of motion (ROM) while crossing their arms over their chest. Three repetitions were carried out at each angular velocity, and the peak torque (PT) value was used. Between each repetition, there was a 30-s rest interval, whereas between the angular velocity assessments, there was a 60-s interval. Throughout the assessment sessions, participants performed maximal voluntary contractions while they were given verbal positive reinforcement during the procedure. Data was gathered at angular velocities of 60, 180, and 300°·s^−1^, and maximal isokinetic strength was identified as the torque of the hamstring and quadriceps muscles when performing movements with a complete range of motion (ROM) [34,35,36].

The ratio between peak values at each angular velocity expresses the concentric strength ratio between knee flexors and extensors muscles. The conventional H:Q ratio was calculated by dividing each participant’s highest concentric peak torque leg flexion by the highest concentric peak torque leg extension.

### 2.3. Statistics

The statistical analysis was performed via SPSS (version 17.0, Chicago, IL, USA) and Microsoft Excel 2013 (Microsoft Corp., Redmond, WA, USA). Data are presented as mean ± SD. The distribution of all dependent variables was examined by the Shapiro–Wilk test and was not found to differ significantly from normal. Analysis of variance was used to analyze the differences among age groups. In order to detect significant differences, a post-hoc Bonferroni test was utilized, along with 95% confidence intervals (CI). Effect sizes for variance analyses were given as partial eta squared (η_p_^2^), with values ≥0.01, ≥0.06, ≥0.14 indicating small, moderate, or large effects, respectively [32]. The level of statistical significance was set at *p* < 0.05. Also, for the strength measurements, the absolute differences (Nm and %) were calculated together with the standard deviation between muscle groups, age groups, and angular velocities, in order to have an appreciation of absolute changes.

## 3. Results

**Figure 1:**
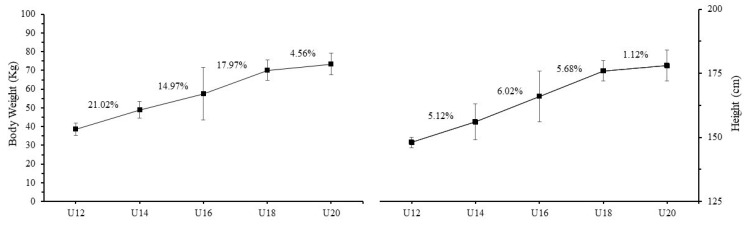
Body weight and height in different age groups (mean ± SD).

The results of the present study showed that the 14-year-olds had a greater (by 21%) body mass compared to the 12-year-olds, directly followed by the 18-year-olds, whose body mass was greater (by 18%) compared to the 16-year-olds. The smallest increase (by 4.6%) was observed in the 20-year-olds compared to the 18-year-olds. Conversely, the greatest increase in height was observed in the 16-year-olds in comparison to the 14-year-olds, at a rate of 6.02% (Figure 1).

**Figure 2:**
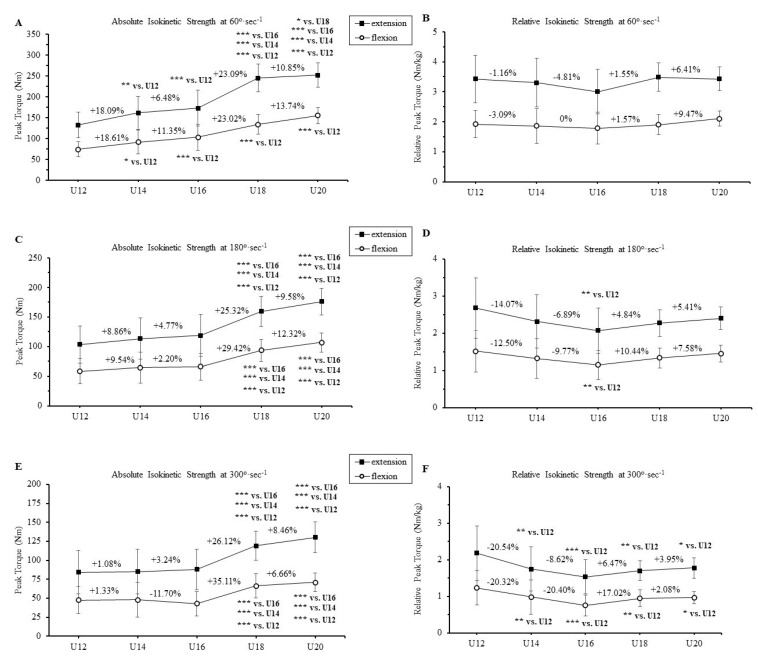
Absolute and relative peak torque values of knee extensors and knee flexors at angular velocities of: (**A**,**B**) 60°·s^−1^; (**C**,**D**) 180°·s^−1^; (**E**,**F**) 300°·s^−1^ in different age groups (mean ± SD). * *p* < 0.05, ** *p* < 0.01, *** *p* < 0.001.

The absolute and relative peak isokinetic strength and the changes (%) of knee extensors and flexors at 60, 180, and 300°·s^−1^ in different age groups are presented in Figure 2. Significant differences were found in absolute isokinetic strength at all angular velocities for the 18-year-old age group as compared to that of the 12-, 14-, and 16-year-old age groups for the knee extensors (*p* < 0.001, respectively; 60°·s^−1^: η_p_^2^ = 0.608; 12-year-olds: CI 87.32 to 166.46, 14-year-olds: CI 116.76 to 204.12, 16-year-olds: CI 126.32 to 219.03; 180°·s^−1^: η_p_^2^ = 0.432, 12-year-olds: CI 70.28 to 137.14, 14-year-olds: CI 74.33 to 151.49, 16-year-olds: CI 79.44 to 158.81; and 300°·s^−1^: η_p_^2^ = 0.352, 12-year-olds: CI 51.23 to 117.12, 14-year-olds: CI 52.33 to 118.46, 16-year-olds: CI 59.45 to 119.09). Similarly, for the knee flexors, significant differences were observed for the angular velocities of 180 and 300°·s^−1^ (*p* < 0.001 respectively), however, at the angular velocity of 60°/s, significant differences were observed only between 12-year-olds and 18-year-olds (*p* < 0.001) (60°·s^−1^: η_p_^2^ = 0.538, 12-year-olds: CI 53.98 to 93.27, 14-year-olds: CI 62.21 to 118.33, 16-year-olds: CI 71.22 to 134.88; 180°·s^−1^: η_p_^2^ = 0.407, 12-year-olds: CI 36.52 to 80.26, 14-year-olds: CI 37.21 to 93.88, 16-year-olds: CI 41.94 to 90.44; and 300°·s^−1^: η_p_^2^ = 0.282, 12-year-olds: CI 27.39 to 68.41, 14-year-olds: CI 22.24 to 73.88, 16-year-olds: CI 25.02 to 41.26). Similar patterns were found in the comparison between the 20-year-olds with the 12-, 14-, 16-, and 18-year-olds for both knee extensors (60°·s^−1^: 12-year-olds: *p* < 0.001, 14-year-olds: *p* < 0.001, 16-year-olds: *p* < 0.001, 18-year-olds: *p* < 0.05, CI 206.97 to 282.01; 180°·s^−1^: 12-year-olds: *p* < 0.001, 14-year-olds: *p* < 0.001, 16-year-olds: *p* < 0.00, 18-year-olds: CI 131.76 to 187.93; and 300°·s^−1^: 12-year-olds: *p* < 0.001, 14-year-olds: *p* < 0.001, 16-year-olds: *p* < 0.001, 18-year-olds: CI 106.59 to 153.09) and knee flexors (60°·s^−1^: 12-year-olds: *p* < 0.001, 18-year-olds: CI 106.91 to 159.84; 180°·s^−1^: 12-year-olds: *p* < 0.001, 14-year-olds: *p* < 0.001, 16-year-olds: *p* < 0.00, 18-year-olds: CI 71.49 to 116.06; and 300°·s^−1^: 12-year-olds: *p* < 0.001, 14-year-olds: *p* < 0.001, 16-year-olds: *p* < 0.001, 18-year-olds: CI 47.41 to 84.58). It is worth noting that no significant differences were found between 14- and 16-year-olds at all angular velocities. At the angular velocity of 60°·s^−1^, significant differences were found for both knee extensors and flexors between the 12- and 14-year-olds (*p* < 0.01 and *p* < 0.05, respectively) and between 12- and 16-year-olds (*p* < 0.001, respectively). It is worth noting that no significant differences were found between 14- and 16-year-olds at all angular velocities. The greatest and most abrupt increases in absolute isokinetic strength were found in the age group of 18-year-olds, as compared to the 16-year-olds for both knee extensors (60°·s^−1^: 23.09%; 180°·s^−1^: 25.32%; 300°·s^−1^: 26.12%) and knee flexors (60°·s^−1^: 23.02%; 180°·s^−1^: 29.42%; 300°·s^−1^: 35.11%). On the contrary, in relative isokinetic strength, significant differences were observed only in the 180 and 300°·s^−1^ between the age groups (180°·s^−1^: 12- vs. 16-year-olds, *p* < 0.01; 300°·s^−1^: 12- vs. 14-year-olds, *p* < 0.01, 12- vs. 16-year-olds, *p* < 0.001, 12 vs. 18-year-olds, *p* < 0.01, 12 vs. 20-year-olds *p* < 0.05) for both knee extensors and knee flexors.

**Figure 3:**
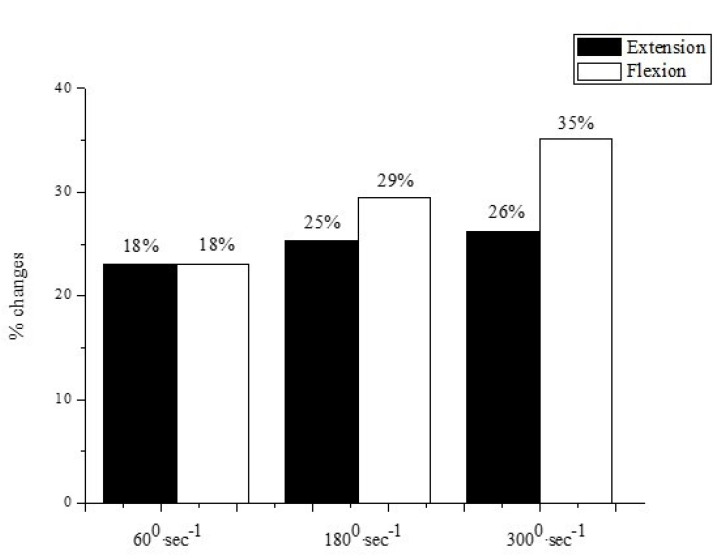
Strength percent increase of knee extension and knee flexion in age group U-18. As angular velocity progressively increased, it was accompanied by a greater strength percent increase in the hamstrings relative to the increase in the quadriceps.

Figure 3 depicts a characteristic example in the U-18 group. At the fast angular velocity of 300°·s^−1^, the increase in hamstrings is greater than the increase in quadriceps (35% vs. 26%, respectively).

The absolute peak torque values of the hamstring and quadriceps muscles, their quantitative differences in Nm, their percent decrease in the peak torque from slower to faster angular velocity, as well as the H:Q strength ratio across angular velocities and age groups, are shown in Table 2. No significant differences were found in the H:Q strength ratio among the angular velocities or between the different age groups. However, in the age group U-12, the greatest H:Q strength ratio appeared at the angular velocity of 180°·s^−1^ (0.56), while in the other age groups, the highest H:Q strength ratio occurred at the angular velocity of 60°·s^−1^. The lowest H:Q strength ratio was observed in the age group U-12 (0.51), while the greatest was observed in the age group U-20 (0.61). Table 2 suggests that when angular velocity was increased progressively from 60 to 300°·s^−1^, strength values of both the hamstring and quadriceps decreased. In the age group U-12, hamstring peak torque decreased from 74.13 to 47.37 Nm (36.09% reduction), while quadriceps peak torque decreased from 144.86 to 84.23 Nm (41.85% reduction). In the U-12 group, at 60°·s^−1^, the value of the quadriceps peak torque was almost twice as much as the hamstrings peak torque value. As age increased, the difference between hamstring and quadriceps peak values decreased. In age group U-14, the decrease in hamstring peak torque from slower to faster angular velocity was 91.09 to 48.01 Nm (47.29% reduction), while the decrease in quadriceps peak torque was from 161.36 to 85.15 Nm (47.22% reduction). Accordingly, the decreases in the other age groups are shown in Table 2.

Examination of correlations between the parameters assessed in our study showed a high correlation between age and peak torque of knee flexion (r = 0.723, *p* < 0.01) and knee extension (r = 0.730, *p* < 0.01) only at the angular velocity of 60°·s^−1^. In addition, high correlations were found between knee flexion and knee extension peak torque at an angular velocity of 60°·s^−1^ (r = 0.875, *p* < 0.01), 180°·s^−1^ (r = 0.856, *p* < 0.01), and 300°·s^−1^ (r = 0.826, *p* < 0.01), respectively. Strong correlations were also observed between body weight and all three angular velocities (60°·s^−1^, r = 0.826, *p* < 0.01; 180°·s^−1^, r = 0.845, *p* < 0.01; 300°·s^−1^, r = 0.830, *p* < 0.01).

## 4. Discussion

The purpose of the present study was to examine the concentric isokinetic strength of the hamstring and quadriceps muscles and their ratio at different angular velocities and chronological ages in young soccer players. It is clearly evident from the results gathered that there is a positive increase in hamstring and quadriceps absolute muscle strength with increasing chronological age across all angular velocities measured. It is essential to mention that there is a link between body weight and height and the reported positive increase, which occurs independently of the participant’s physique while they are growing. However, relative muscle strength follows the opposite pattern, namely, it decreases with increasing chronological age. As expected, body height and body weight increased linearly with chronological age.

Teenagers at the age of 14–16 years go through a major transition, moving from their childhood to their adult life; this is a period that is also characterized by their sexual maturation as well as body growth [37]. First, Tanner [38], followed by Beunen and Malina [39], observed a high degree of complexity in the changes occurring throughout these years of transition in terms of biological growth, which are also related to the nervous and endocrine systems of teenagers. They also reported anthropometric and physiological changes occurring during the same period. Among the findings of this study, a high level of body mass increase was observed in the 14-year-old participants, which can be the result of their rapid body and height growth, typical of their age. During the age of 14–16 years, resistance and strength are also improved, and training can be a stimulus for increased growth hormone and testosterone levels [40].

Previous research demonstrated similar values of the H:Q conventional ratio for 60°·s^−1^ [41,42], 180°·s^−1^ [43,44], and 300°·s^−1^ [44]. However, greater values were observed in these angular velocities, ranging between 60 to 83% [24,45]. There are several studies with similar samples, but the examined angular velocities were different [46,47].

In general, the torque–velocity relationship in young populations was observed to have a pattern similar to that of the adult population, which is expressed by the inverse relationship between angular velocity and peak torque: the former increases while the latter decreases. Quadriceps strength was greater than hamstring strength at all angular velocities measured and across all age groups, which is in agreement with all previous studies that have investigated quadriceps and hamstring strength with an isokinetic dynamometer. In the present study, the smallest H:Q strength ratio was found in the youngest age group (U-12 = 0.51) and the greatest H:Q strength ratio in the eldest age group (U-20 = 0.61). This by no means suggests that there is a linear relationship between the H:Q strength ratio and age. For example, the H:Q strength ratio was greater in the age group U-16 than U-18.

In age group U-12, the quadriceps muscle strength at 60°·s^−1^ was almost twice that of the hamstring muscle strength. Interestingly, as age increased, this difference decreased. This may be due to improved neuromuscular function and the adaptations resulting from the higher workout intensity, which applies to older ages. This age group displayed the greatest H:Q strength ratio at a moderate angular velocity. It is possible that muscles in this age group have the ability to recruit available motor units more efficiently at a moderate angular velocity when the resistance is smaller than at a slower angular velocity.

It is common for soccer players in the age groups U-18 and U-20 to move up from youth competition to adult competition. In these new conditions, they undergo more systematic strength and endurance training, which leads to hormonal and metabolic adaptations [40]. Age and training intensity seem to play an important role in soccer performance. Players at a high level of competition and skill have greater concentric isokinetic peak torque values in the quadriceps and hamstring muscle groups as well as a greater H:Q strength ratio [28,48]. Recent studies of these ages had mean values of 50–83% at slow angular velocities, 51–80% at intermediate velocities, and 50–89% at fast velocities. However, some studies that examined different angular velocities did not clearly show an effect of velocity on H:Q ratio [20,24,43,45,48,49]. Our results are in agreement with Dauty et al. [45], with an expressive sample size (n = 136), who reported an increase in H:Q conventional ratios at higher angular velocities.

The quadriceps and hamstrings are two muscle groups with different functions. It has been reported that co-activation of hamstring muscles significantly increases during concentric quadriceps muscle contraction. However, this is not the case for the opposite, that is, co-activation of quadriceps muscles does not change during concentric hamstring muscle contraction [50]. Sangnier and Tourny–Chollet [18], who investigated a population of soccer players during isokinetic endurance testing, showed a divergence in fatigue resistance between the quadriceps and hamstring muscles in concentric mode. The significant decline in hamstring strength was shown to affect the balance of strength between the agonist and antagonist muscles.

A critical view of this study’s findings on the relationship between anterior and posterior thigh muscle strength by no means challenges the widely accepted view that when these muscles have adequate and balanced strength, they stabilize joints, protecting them from injury. As far as the young soccer players are concerned, the data gathered on the H:Q ratio during this study may not be helpful enough regarding the development of a tool for the prevention of hamstring or quadriceps muscle injuries. On the other hand, when it comes to adult soccer players, the findings differ. In order for them to perform at their best, a higher level of muscle strength and endurance is required. In this case, a high H:Q ratio is not only essential but it may also have positive results; this H:Q value is likely to prevent knee joint injuries when heavy forces are exerted on it. In this study, it is supported that there might be a connection between lower extremity injuries and the particularly small H:Q strength ratio [14,51,52].

When assessing the players’ training on the pitch, it was evident that the hamstring muscles were not stimulated regularly, nor were they trained with eccentric exercises. During their training with additional weights, soccer players did not focus on training the hamstring muscles, but on performing mostly flexion–extension exercises for the knee joint. In addition, hamstring muscles are more difficult to stimulate than quadriceps muscles, regardless of the extent of training; it is the positions and starting points of the training that result in this variation. Despite the emphasis during training, which stresses the fact that knee flexor strength is extremely important in soccer players for joint stabilization during various tasks, it seems that strength training of the hamstring muscles remains inadequate. To conclude, it is evident that there is an inverse connection between the H:Q strength ratio and angular velocity, i.e., as angular velocity increases, the H:Q ratio decreases.

### Limitations and Future Directions

This study had several limitations. The player position was not considered for analysis; therefore, the findings of the study cannot be specialized according to the position roles of the soccer players (goalkeepers, defenders, midfield players, and attackers). Also, the participants came from the same soccer academy and followed a specific soccer training program, so the conclusions should not be generalized. Finally, despite the large number of participants, the biological age was not examined; therefore, the effects of growth and development may have affected the results of the study. Future analyses could embrace areas such as the players’ positions and the participants’ biological age, especially during the developmental ages (12- to 16-year-olds), and accompany the laboratory measurements with practical tests on the field.

## 5. Conclusions

In conclusion, the present study indicates that quadriceps muscle strength is greater than that of its antagonist muscle group, and there is an inverse connection between H:Q strength ratio and angular velocity, i.e., as angular velocity increases, the H:Q ratio decreases. Also, the small H:Q strength ratio in younger ages and the large H:Q ratio in older ages suggest that higher volume and intensity training may increase the H:Q strength ratio, which, in turn, may protect the knee joint from excessive and burdensome loads. The findings of the present study could be used as normative data for soccer players regarding the strength evaluation of the hamstring and quadriceps muscles using an isokinetic dynamometer. This knowledge can be used as a springboard for more efficient strengthening of the involved muscles. Although this study provides important information regarding the H:Q strength ratio’s development with age, a longitudinal study design would provide even more valid data.

## Figures and Tables

**Table 1:** Physical characteristics of the subjects (mean ± SD).

	U-12 n = 43	U-14 n = 63	U-16 n = 64	U-18 n = 53	U-20 n = 42
**Age (yrs)**	11.50 (±0.40)	13.60 (±0.30)	15.40 (±0.50)	17.50 (±0.40)	19.30 (±0.60)
**Training age (yrs)**	3.2 (±0.60)	5.50 (±1.00)	7.40 (±2.00)	8.50 (±1.50)	10.90 (±1.90)
**Height (cm)**	148 (±2.04)	156 (±7.02)	166 (±1.01)	176 (±4.02)	178 (±6.02)
**Weight (kg)**	38.61 (±3.31)	48.89 (±4.48)	57.50 (±14.05)	70.10 (±5.45)	73.45 (±5.80)

**Table 2:** Absolute peak torque values of the knee extensors and knee flexors, their quantitative differences expressed in Nm, their percent decrease from slower to faster angular velocity, and the H:Q strength ratio across angular velocities and age groups (mean ± SD).

U12				Decrease%		
**Angular velocity**	**60·s^−1^**	**180·s^−1^**	**300·s^−1^**	**60–180**	**180–300**	**60–300**
**Hamstrings**	74.13 (±17.59)	58.46 (±21.30)	47.37 (±17.96)	21.13	18.97	36.09
**Quadriceps**	144.86 (±30.48)	103.51 (±31.33)	84.23 (±28.75)	28.54	18.62	41.85
**Differences** **(Nm)**	−70.73	−44.5	−36.86	−7.41	0.35	−5.76
**H:Q (%)**	51.17	56.47	56.07			
**U14**				**Decrease %**		
**Angular velocity**	**60·s^−1^**	**180·s^−1^**	**300·s^−1^**	**60–180**	**180–300**	**60–300**
**Hamstrings**	91.09 (±28.30)	64.63 (±26.02)	48.01 (±22.81)	29.04	25.71	47.29
**Quadriceps**	161.36 (±39.61)	113.28 (±35.01)	85.15 (±29.41)	29.79	24.83	47.22
**Differences** **(Nm)**	−70.27	−48.65	−37.14	−0.75	0.88	0.07
**H:Q (%)**	59.89	57.82	59.82			
**U16**				**Decrease %**		
**Angular velocity**	**60·s^−1^**	**180·s^−1^**	**300·s^−1^**	**60–180**	**180–300**	**60–300**
**Hamstrings**	102.76 (±31.01)	66.09 (±22.62)	42.98 (±16.00)	35.68	34.96	58.17
**Quadriceps**	172.53 (±42.65)	118.96 (±35.07)	88.01 (±26.67)	31.04	26.01	49.07
**Differences** **(Nm)**	−69.77	−47.13	−45.03	4.64	−8.95	9.10
**H:Q (%)**	59.56	55.55	48.83			
**U18**				**Decrease %**		
**Angular velocity**	**60·s^−1^**	**180·s^−1^**	**300·s^−1^**	**60–180**	**180–300**	**60–300**
**Hamstrings**	133.49 (±23.75)	93.64 (±18.60)	66.24 (±15.94)	29.85	29.26	50.37
**Quadriceps**	224.33 (±33.38)	159.30 (±25.28)	119.13 (±18.89)	29.98	25.21	46.89
**Differences** **(Nm)**	−90.84	−65.66	−52.89	−0.13	4.05	3.48
**H:Q (%)**	59.50	58.78	55.60			
**U20**				**Decrease %**		
**Angular velocity**	**60·s^−1^**	**180·s^−1^**	**300·s^−1^**	**60–180**	**180–300**	**60–300**
**Hamstrings**	154.76 (±18.91)	106.80 (±16.46)	70.97 (±12.21)	30.98	33.54	54.14
**Quadriceps**	251.66 (±28.76)	176.19 (±22.40)	130.14 (±20.19)	29.98	35.93	48.28
**Differences** **(Nm)**	−96.90	−69.39	−57.98	1.00	−2.39	5.86
**H:Q (%)**	61.49	60.61	54.53			

## Data Availability

The data presented in this study are available on request from the corresponding author. The data are not publicly available due to privacy restrictions.
